# Comprehensive microsurgical anatomy of the middle cranial fossa: Part II—neurovascular anatomy

**DOI:** 10.3389/fsurg.2023.1132784

**Published:** 2023-03-24

**Authors:** Ali Tayebi Meybodi, Giancarlo Mignucci-Jiménez, Michael T. Lawton, James K. Liu, Mark C. Preul, Hai Sun

**Affiliations:** ^1^ Department of Neurosurgery, Rutgers New Jersey Medical School, Newark, NJ, United States; ^2^ The Loyal and Edith Davis Neurosurgical Research Laboratory, Department of Neurosurgery, Barrow Neurological Institute, St. Joseph’s Hospital and Medical Center, Phoenix, AZ, United States; ^3^ Department of Neurosurgery, Barrow Neurological Institute, St. Joseph’s Hospital and Medical Center, Phoenix, AZ, United States; ^4^ Departments of Neurosurgery and Otolaryngology, Robert Wood Johnson Barnabas Health, Newark, NJ, United States; ^5^ Department of Neurosurgery, Robert Wood Johnson Medical School, Rutgers University, New Brunswick, NJ, United States

**Keywords:** geniculate ganglion, internal carotid artery, laterotrigeminal venous system, greater superficial petrosal nerve, lesser petrosal nerve, middle meningeal artery, petrous bone

## Abstract

In order to master the surgical approaches to the middle cranial fossa, the surgeon needs to understand the relevant bony anatomy. However, she/he also needs to have a clear and sound understanding of the neural and vascular anatomy because, oftentimes, the osseous anatomy (except for the optic apparatus) should be removed to expose and protect the neurovascular anatomy. This is the second of a two-part article discussing the neurovascular anatomy of the middle cranial fossa. A brief discussion of the surgical approaches follows.

## Introduction

The middle cranial fossa (MCF) houses several important structures such as the otic apparatus and the internal carotid artery (ICA). A proper understanding of the detailed surgical anatomy of this region is critical when using approaches involving the middle fossa. This is the second of a 2-part article that focuses on the microsurgical anatomy of the neurovascular structures and briefly discusses the surgical approaches to the middle fossa.

## Arterial anatomy

Two major arteries (and their branches) deserve discussion in the context of MCF anatomy: (1) ICA and (2) middle meningeal artery (MMA).

### Internal carotid artery

**Figure 1:**
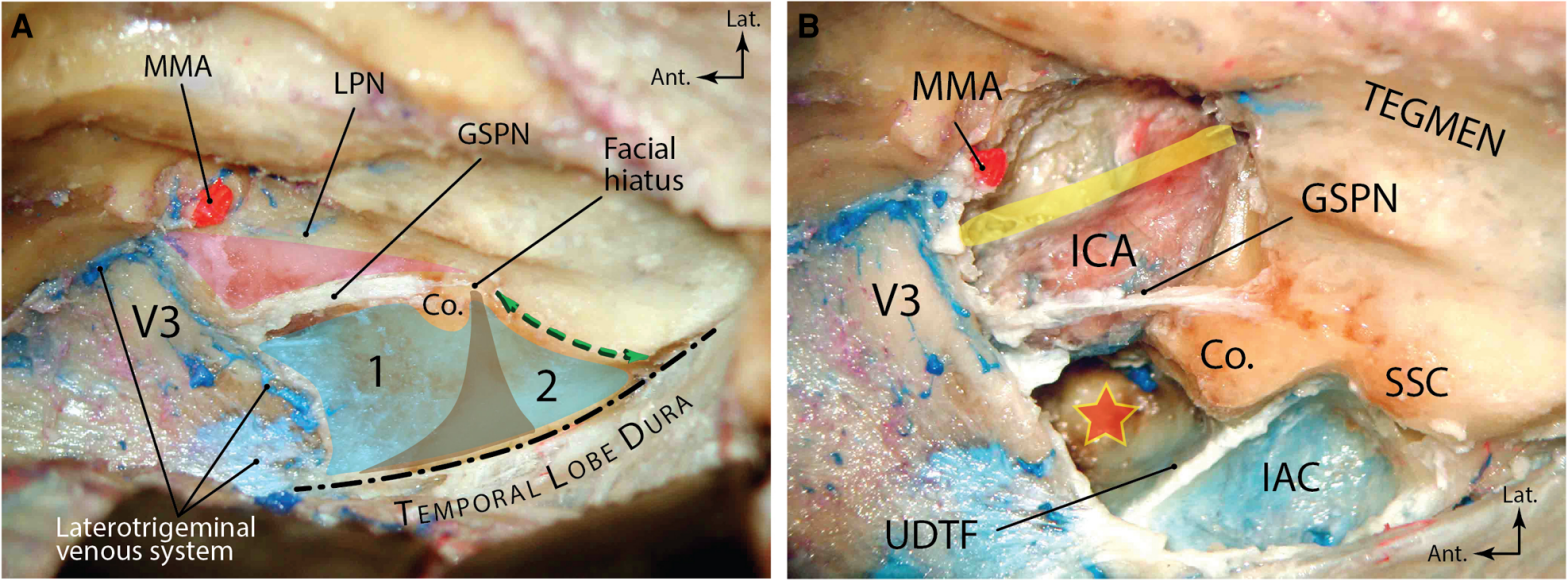
Overview of middle fossa anatomy (right side). (**A**) The temporobasal dura has been elevated to expose the neurovascular anatomy of the middle fossa floor. Blue area shows the middle fossa rhomboid divided by the funnel-shaped internal auditory canal (brown area) into an anterior (premeatal) triangle (1) and a posterior (postmeatal) triangle (2). Pink region denotes the Glasscock triangle. Green double-arrow shows the arcuate eminence and the trajectory of the underlying superior semicircular canal. Dashed line shows the medial petrous ridge exposed by elevation of the temporobasal dura. (**B**) Exposure of the underlying neurovascular structures after drilling the Kawase and Glasscock triangles. Yellow band shows the direction of the Eustachian tube. Red star shows the drilled out petrous apex. Ant., anterior; Co., cochlea; GSPN, greater superficial petrosal nerve; IAC, internal auditory canal; ICA, internal carotid artery; lat., lateral; LPN, lesser petrosal nerve; MMA, middle meningeal artery; SSC, superior semicircular canal; UDTF, upper dural transitional fold. (From Zhao and Liu. *Neurosurg Focus*. 2008 **25**(6): E5. Used with permission from JNS Publishing Group.)

**Figure 2:**
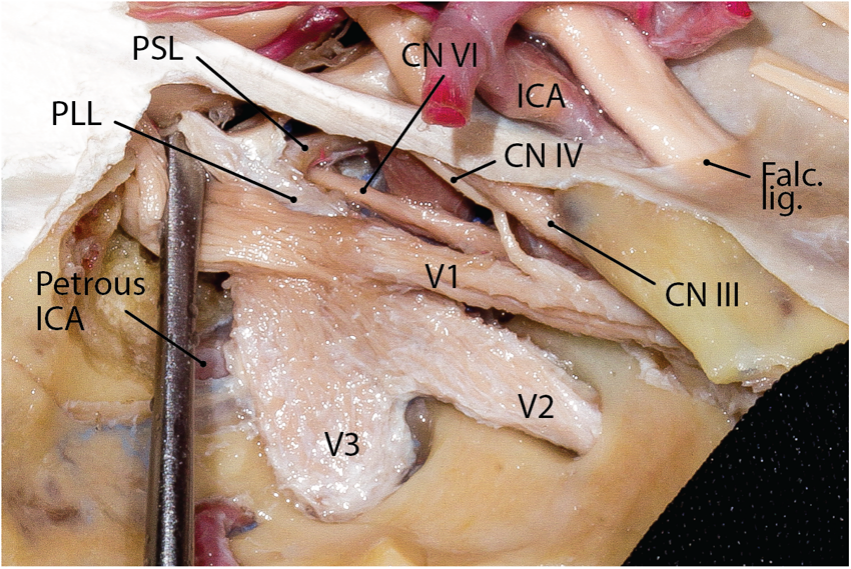
Transition of the petrous ICA to the cavernous ICA under the trigeminal nerve. Note the relationship between the petrolingual and petrosphenoid ligaments. CN, cranial nerve; ICA, internal carotid artery; lig., ligament; PLL, petrolingual ligament; PSL, petrosphenoid ligament. (Courtesy of Barrow Neurological Institute, Phoenix, AZ, United States. Used with permission*.*)

The petrous ICA runs under the middle fossa floor in the carotid canal. It courses anteromedially almost parallel to the petrous ridge. Upon reaching the petrous apex, it makes an upward turn under the trigeminal ganglion (separated from the nerve by the petrolingual ligament) before entering the cavernous sinus (Figure 1). The petrolingual ligament runs between the petrous apex and the lingual process of the sphenoid bone (see Part 1). It should be distinguished from the petrosphenoid ligament as the vault of Dorello's canal (Figure 2). The petrous ICA is encased by the sympathetic plexus and embedded in a delicate venous plexus. Up to 70% of specimens are reported to have branches coming from the petrous ICA (1). Branches of the petrous ICA include (1) the caroticotympanic artery, (2) the mandibular artery, (3) the vidian artery, and (4) periosteal arteries.

#### Caroticotympanic artery

Reportedly, the caroticotympanic artery emanates from the posterior wall of the ICA genu between the posterior vertical and horizontal segments (2). It enters the cavity of the middle ear to feed the cavity and anastomoses with the tympanic branch of the ascending pharyngeal artery (3). It is considered a remnant of the hyoid stapedial artery (3). In some studies, no caroticotympanic branch was found (4).

A rare (yet important) branch of the petrous ICA is the persistent embryonic stapedial artery. Originating from the dorsal aspect of the first vertical segment of the petrous carotid, this artery initially serves to give rise to branches that eventually emanate from the internal maxillary artery (IMA) (i.e., inferior alveolar, infraorbital, middle meningeal, anterior ethmoidal, frontal, supraorbital, and lacrimal arteries) before it involutes (5). If the stapedial artery persists, the MMA will arise from it with the foramen spinosum (FS) being absent (1:5,000) (6). After originating from the ICA, the artery enters the hypotympanum and makes a dorsally convex curve passing through the obturator foramen of stapes. It finally exits the middle ear cavity through entering the labyrinthine facial canal end enters the middle fossa through the facial hiatus (6, 7).

#### Mandibular artery

The mandibular artery is a remnant of an embryonic branch arising from the horizontal segment of ICA. It gives rise to two branches directed anteriorly: (a) The artery of the pterygoid canal (also known as vidian artery), which runs in the pterygoid canal, anastomosing with the vidian branch of the internal maxillary artery either in the pterygopalatine fossa or in the vidian canal. (b) The second branch courses more caudally and anastomoses with the pterigovaginal artery near the Eustachian tube (8).

#### Vidian and periosteal arteries

In some descriptions, the petrous ICA is considered to lack a mandibular branch and instead directly giving off the vidian and periosteal branches following the caroticotympanic artery (1, 4). These arteries arise near the anterior genu of the petrous ICA. The vidian artery courses anteriorly and medially to reach superior and lateral to the cartilage of the foramen lacerum to enter the posterior opening of the vidian canal along with the vidian nerve. The periosteal arteries may be more than one (average of 1.5 branches per specimen) and in 45% of specimens have a common trunk with the vidian artery. These arteries supply the periosteum of the carotid canal and some may reach the dura of the middle fossa (1).

### Middle meningeal artery

**Figure 3:**
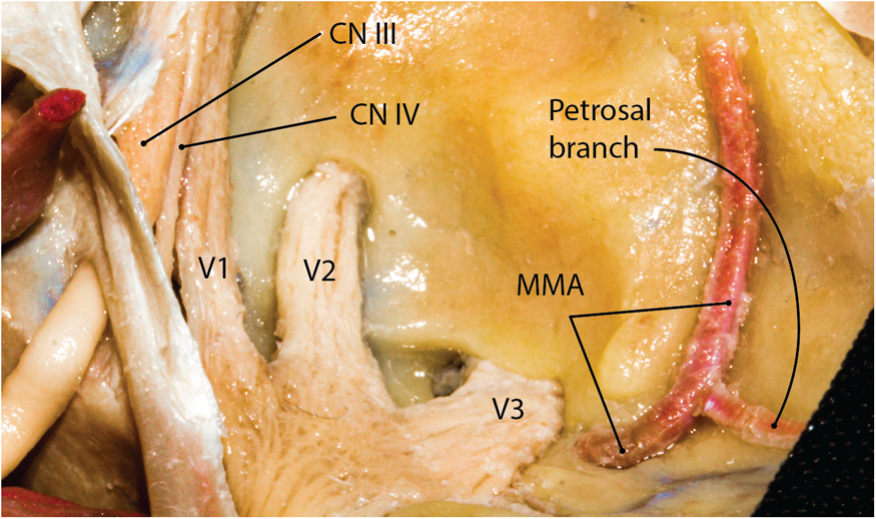
Middle meningeal artery on the floor of the middle fossa (right side). The petrosal branch is the first branch of the MMA before it gives rise to anterior and posterior divisions that feed the convexity dura. CN, cranial nerve; MMA, middle meningeal artery. (Courtesy of Barrow Neurological Institute, Phoenix, AZ, United States. Used with permission*.*)

**Figure 4:**
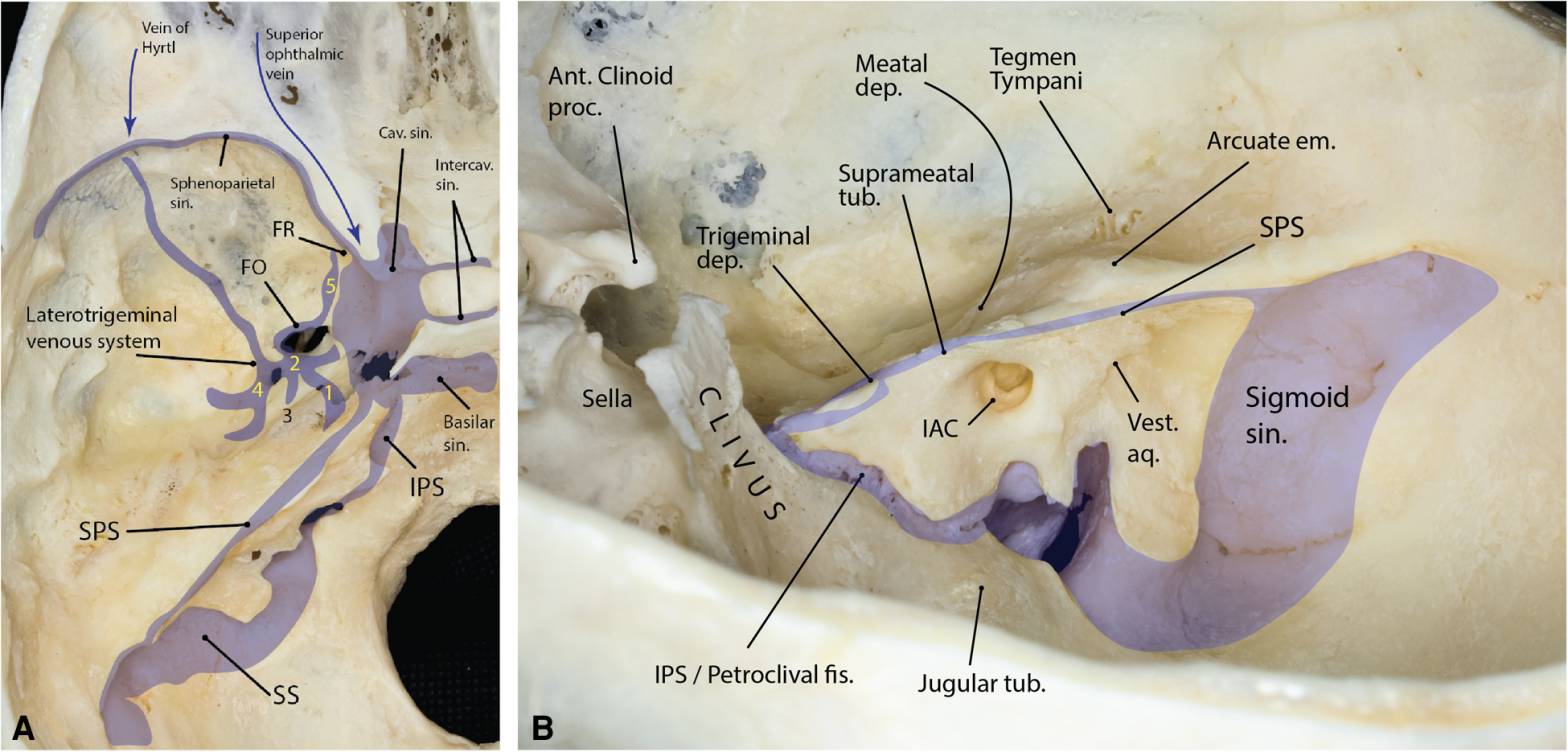
(**A**) Summary of the major venous compartments of the middle fossa (left side). The laterotrigeminal system and its compartments are shown. (1) venous canal on the trigeminal nerve root and lateral aspect of the Gasserian ganglion; (2) venous lacuna on the lateral border of V3; (3) communication to the petrosal nerve venous lake; (4) middle meningeal vein around the foramen spinosum; (5), emissary vein of the foramen ovale extending anteriorly toward foramen rotundum. (Adapted from Simões. An anatomical study of the laterotrigeminal venous system. *Ann Anat*. 1993;**175**(2):115–8) (**B**) Relationship between the petrous face and major bony landmarks of the middle cranial fossa (right side). The inferior petrosal sinus marks the inferior boundary of the Kawase triangle drilled during the middle fossa approach. Note the relationship between the arcuate eminence, suprameatal tubercle and the trigeminal depression. ant., anterior; aq., aqueduct; cav., cavernous; dep., depression; em., eminence; fis., fissure; IAC, internal auditory canal; IPS, inferior petrosal sinus; proc., process; sin., sinus; SPS, superior petrosal sinus; SS, sigmoid sinus; tub., tubercle; vest., vestibular. (Copyright Ali Tayebi Meybodi. Used with permission.)

The MMA could originate from the IMA, ICA, basilar artery, or ophthalmic artery. However, most frequently, the MMA arises from the superior aspect of the first (mandibular) part of the IMA after the deep auricular artery (entering the squamotympanic fissure) and the anterior tympanic artery (which enters petrotympanic fissure) (See Figure 4 in Part 1). It enters FS after passing posterior to the condylar process of the mandible. Once in the MCF, the MMA grooves the greater sphenoid wing on the floor of the MCF making an anteriorly convex curve when viewed laterally. Immediately after coursing through the FS, a small trunk originates from the MMA that rapidly divides into anterior and posterior branches (Figure 3). The posterior branch is the *petrosal arter*y (less than 1 mm diameter), which runs posterolaterally [while being lateral to the lesser petrosal nerve (LPN)] toward the facial hiatus or a laterally located bony canal in the MCF. According to El-Khouly et al., the petrosal artery can is present in 80% of specimens. When present, the petrosal artery can arise from the MMA either above (81%) or below (19%) the FS (9). The petrosal artery supplies the greater superficial petrosal nerve (GSPN), LPN, geniculate ganglion, and facial nerve. The anterior branch is the *cavernous branch of the MMA* and courses anteromedially to reach the lateral aspect of the Gasserian ganglion and eventually anastomosing with the inferolateral trunk (a branch of the cavernous ICA). It gives rise to anterior and posterior divisions as it ascends laterally to reach the temporoparietal dural convexity. The point of division is usually 15–30 mm anterolateral to the FS. The anterior division further divides into medial and lateral branches. The lateral branch reaches the pterion while the medial branch courses beneath and parallel to the lesser sphenoid wing finally anastomosing with the recurrent meningeal branch of the lacrimal artery (10). As mentioned before, the MMA rarely originates from a persistent stapedial artery. The posterior division of the MMA supplies the dura of the convexity and gives two branches: (1) petrosquamosal and (2) parieto-occipital. The petrosquamosal branch runs low along the junction of the temporal bone squama and the MCF to supply the lateral and posterior dura of the MCF near and around the transverse sinus.

## Venous anatomy

The MCF is like a shallow venous basin located lateral to the cavernous sinus with robust connections to the rest of the skull base venous channels. The potential space between the meningeal and endosteal layers of the dura contains venous spaces in different areas of the MCF. This potential space is prominent in the lateral aspect of the trigeminal ganglion (i.e., the laterotrigeminal venous system), anteriorly, along the sphenoid wing (sphenoparietal sinus), and medially along the petrous pyramid [superior petrosal sinus (SPS)]. The superior ophthalmic vein (SOV) runs through the superior orbital fissure (SOF) and is briefly discussed. The SPS is the medial venous sinus of the MCF connecting the cavernous and transverse-sigmoid sinuses (Figure 4).

### Superior ophthalmic vein

The SOV is the largest orbital vein. It originates in the anteromedial orbit medial to the superior rectus muscle insertion. Coursing posterolaterally between the superior rectus muscle and the optic nerve, it reaches the SOF at its lateral half outside the common annular tendon. In the SOF, it runs between the frontal nerve medially and lacrimal nerve laterally. It drains into the anterior compartment of the cavernous sinus posteriorly.

### Sphenoparietal sinus

**Figure 5:**
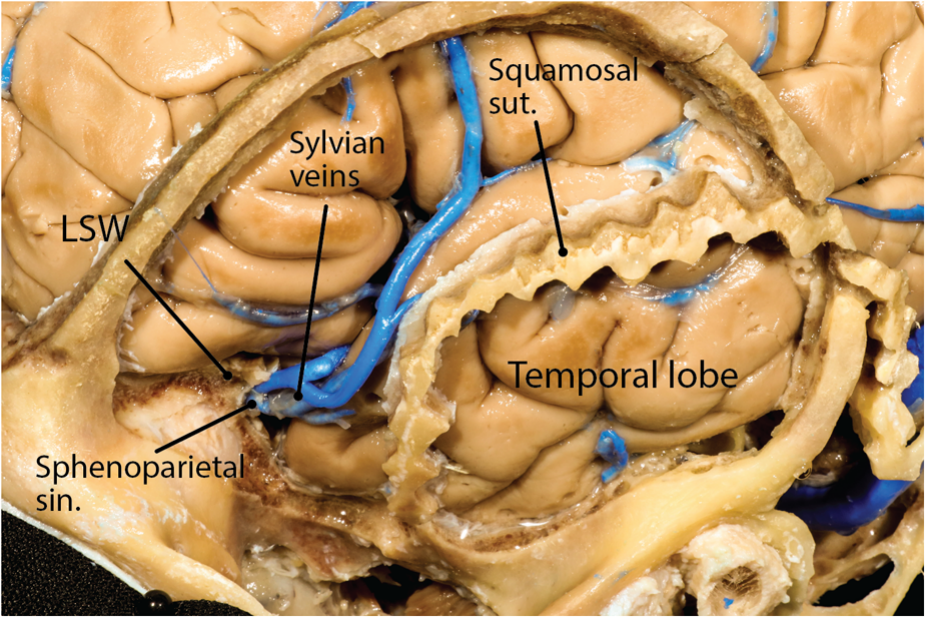
Sphenoparietal sinus under the lesser sphenoid wing and receiving the Sylvian vein. LSW, lesser sphenoid wing; sin., sinus; sut., suture. (Courtesy of Barrow Neurological Institute, Phoenix, AZ, United States. Used with permission.)

The MMA is accompanied by a venous channel between the two layers of the dura. Specifically, the anterior division of the MMA is accompanied by a robust venous space above the level of the pterion. This venous space deviates away from the MMA near the sphenoid wing and turns medially under the lesser sphenoid wing at the temporal tip to join the cavernous sinus. The venous compartment under the lesser sphenoid wing and its superolateral continuation toward parietal convexity has been traditionally called the sphenoparietal sinus after this term was coined by Gilbert Breschet (French anatomist, 1784–1845) (Figure 5) (11). Commonly, the sphenoparietal sinus receives the superficial Sylvian vein. Other tributaries include (1) a diploic vein of the orbital roof, (2) a diploic vein located within the greater sphenoid wing and draining into the pterygoid plexus, and (3) an orbital vein corresponding to the ophthalmo-meningeal vein (of Hyrtl) (Figure 4) (12). Less commonly (12.5% of cases), the sinus turns posteriorly to run in the basal dural of the temporal lobe and empty to the pterygoid venous plexus through the MCF foramina in which case it is called the sphenobasal sinus (13). Alternatively, in 4% of cases, it runs even more posteriorly and joins the sigmoid-transverse junction in which case it is called the sphenopetrosal sinus (13, 14).

### Laterotrigeminal venous system

This is a system of venous spaces centered along the lateral edge of the trigeminal nerve root as it enters the MCF and lateral border of the Gasserian Ganglion between the two layers of the dura of the temporal base. It continues posteriorly to connect with the SPS in 94% of cases (15). Posteriorly, it joins small venous channels along the GSPN and around the FS. It extends anteriorly under the V3 as “the subtrigeminal mandibular communicating sinus” and the emissary sinus of foramen ovale (FO) on the anterolateral surface of the mandibular nerve. In 30% of cases, it extends further along the lateral border of the V3 to reach the FO. It communicates with the pterygoid venous plexus through the emissary vein of FO (15).

### Superior petrosal sinus

The SPS runs along the medial ridge of the petrous part of the temporal bone from the cavernous sinus to the junction of the transverse and sigmoid sinuses. The SPS may be “complete” (i.e., connected both anteriorly to the cavernous sinus and posteriorly to the transverse-sigmoid junction—60% prevalence), “lateral” (i.e., only connected to the transverse-sigmoid junction—37% prevalence), or “medial” (i.e., only connected to the cavernous sinus—3% prevalence) (16). Once close to the trigeminal root at the entrance of Meckel's cave, it may form a venous ring around it (∼15%), or run above (∼65%) or under it (∼20%) (17, 18). The most prominent tributary of the SPS is the superior petrosal vein (aka vein of Dandy). The point of entry of the vein of Dandy to the SPS varies but is most commonly between the internal auditory canal (IAC) and the point of entry of the trigeminal nerve root to Meckel's cave (19).

## Neural anatomy

The MCF is a conduit for several nerves either on its floor or through the foramina and canals. Understanding the microanatomy of these nerves and their three-dimensional relationship with each other and adjacent structures is critical when performing surgical approaches in this region.

### Trigeminal nerve

**Figure 6:**
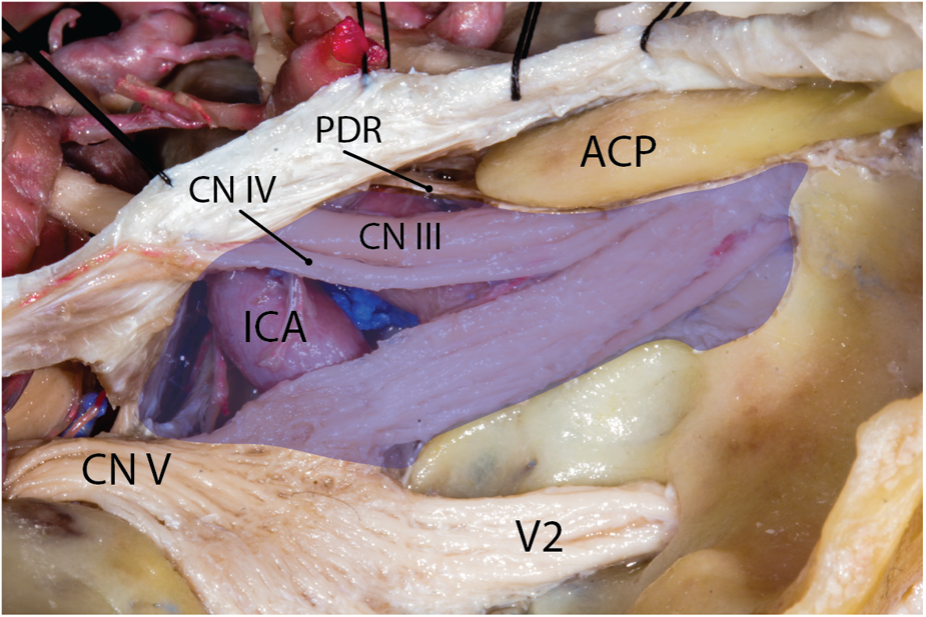
Cavernous sinus and the trigeminal nerve divisions. Blue area shows the silhouette of the cavernous sinus. Note that V2 is not in the venous space of the cavernous sinus. ACP, anterior clinoid process; CN, cranial nerve; ICA, internal carotid artery; PDR, proximal dural ring. (Courtesy of Barrow Neurological Institute, Phoenix, AZ, United States. Used with permission*.*)

Meckel's cave ostium lies inferior to the SPS on the trigeminal depression of the petrous bone a few millimeters posterior to the petrous apex. It is also located just lateral to the posterior end of the cavernous sinus at the petrous apex region. The cisternal segment of the trigeminal root enters the opening of Meckel's cave and rapidly expands to form the Gasserian (aka semilunar) ganglion inferolateral to the cavernous sinus while both structures are covered with the meningeal layer of the dura of the temporal lobe (i.e., dura propria) (see cavernous sinus and Figure 6 in Part 1). Importantly, a small superomedial portion of the Gasserian ganglion is covered laterally by the posteroinferior tail of the cavernous sinus (Figure 6) (20). The subarachnoid space extends roughly to the mid-portion of the Gasserian ganglion through the Meckel's cave ostium. The petrous ICA makes an upward turn near the petrous apex to exit the carotid canal and courses under the trigeminal nerve before entering the cavernous sinus. The petrolingual ligament, extending between the lingual process of the sphenoid bone and the lateral cornu of the petrous apex separates the ICA from the trigeminal nerve just before entering the cavernous sinus (Figure 3). The three divisions enter their respective foramina to exit the MCF.

#### Ophthalmic nerve (V1)

**Figure 7:**
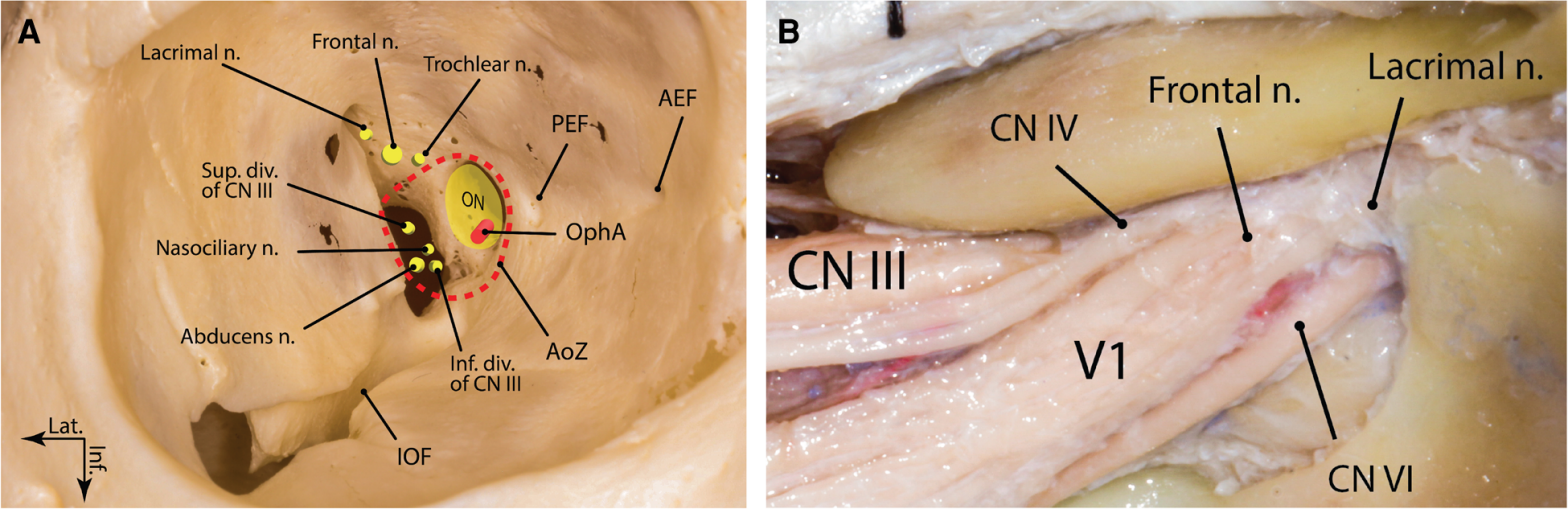
Superior orbital fissure: (**A**) bony anatomy; (**B**) neural anatomy (right side). AEF, anterior ethmoidal foramen; AoZ, annulus of Zinn; CN, cranial nerve; div., division; Inf., inferior; IOF, inferior orbital fissure; Lat., lateral; n., nerve; ON, optic nerve; OphA, ophthalmic artery; PEF, posterior ethmoidal foramen; Sup., superior. (Courtesy of Barrow Neurological Institute, Phoenix, AZ, United States. Used with permission.)

The V1 is the smallest division of the CN V and contains somatic sensory, parasympathetic fibers from the pterygopalatine ganglion and sympathetic fibers from the plexus around the carotid artery. From the CN V ganglion, the V1 is inclined upward as it passes forward in the lower part of the lateral wall of the cavernous sinus to reach the superior orbital fissure. As the ophthalmic nerve approaches the SOF, it branches into the lacrimal, frontal, and nasociliary nerves (21). The lacrimal and frontal branches pass through the lateral sector, and the nasociliary branch passes through the central sector of the SOF (22). At the SOF, the lacrimal nerve is the most lateral nerve, while coursing above the superior ophthalmic vein. The distance between the lacrimal nerve and the lateral end of the SOF is 4.2 ± 1.7 mm. On the other hand, the nasociliary nerve gently ascends lateral to the inferior division of the oculomotor nerve while passing through the fissure, on average 11.3 ± 2.8 mm from the lateral end of the SOF (23). The inferior border of the nasociliary nerve usually gives rise to the sensory root of the ciliary ganglion in the cavernous sinus while coursing between the abducens nerve laterally and inferior division of the oculomotor nerve medially. The frontal nerve is the largest branch of the V1 division and passes through the SOF medial to the superior ophthalmic vein and the lacrimal nerve and inferior to the trochlear nerve, on average 9.3 ± 1.7 mm medial to the lateral end of the SOF (Figure 7) (23).

#### Maxillary nerve (V2)

The V2 is the intermediate division of the CN V and looks like a flattened band. Unlike both V1 and V3, it has only sensory functions. Contrary to many classic textbooks, more recent views hold that V2 does not course in the lateral wall of the dural envelop of the cavernous sinus, as does V1 (24). The V2 exits the middle cranial fossa through foramen rotundum (FR) to enter the pterygopalatine fossa. The laterotrigeminal venous system, which is the venous system surrounding the trigeminal nerve root and the lateral aspect of V3, may extend to the region of the foramen rotundum and lateral aspect of the V2 (Figure 4A). The only branch off the V2 in the MCF is the middle meningeal branch, which is the smallest V2 branch. It arises near the FR and receives a ramus from the ICA sympathetic plexus. Accompanied by the frontal branch of MMA, it supplies the dura mater of the MCF. Its anterior small branches reach the anterior cranial fossa (25).

#### Mandibular division (V3)

The V3 is the largest division of the trigeminal nerve containing sensory, motor, and parasympathetic fibers to the salivary glands. The V3 contains efferent fibers that innervate muscles that are attached to the mandible. It is made up of two roots: a large, sensory root, which proceeds from the lateral part of the CN V ganglion and emerges through FO and a small motor root arising around the superomedial part of the main sensory root in the pons that passes below the trigeminal ganglion and unites with the sensory root just outside FO (24). The V3 exits the MCF through FO to enter the infratemporal fossa.

### Greater superficial petrosal nerve

The GSPN, also known as the greater petrosal nerve, is a branch of the nervus intermedius (nerve of Wrisberg). Nervus intermedius carries parasympathetic fibers, nerve fibers for taste, and sensory fibers of the facial nerve (CN VII) (26). The GSPN contains the parasympathetic fibers originating from the superior salivatory nucleus in the brainstem reaching the pterygopalatine ganglion. It also contains taste afferents from the soft palate (to the gustatory nucleus of the solitary tract in brainstem) and sensory afferents from the dura mater and ICA to the spinal nucleus of trigeminal nerve (27). The sensory fibers pass through the ganglion and continue to the nasal cavity and palate. It is the first branch of the CN VII that arises from the geniculate ganglion. It innervates the lacrimal glands and mucous membranes of the nasal cavities and the palate as major secretory fibers. Once it arises from the geniculate ganglion, it courses anteromedially to leave the superior surface of the temporal bone through the facial hiatus and into the MCF (28). Then, the nerve courses anteromedially on the floor of the MCF, medial to LPN and lateral to ICA. It passes beneath the V3 and toward the foramen lacerum and the pterygoid canal (see Part 1, Figures 5A, 6E) (29). The point where the GSPN crosses beneath V3 is approximately 7.5 mm posteromedial on the lateral aspect of the V3 from FO (range 3–12 mm) (30). It finally joins with the deep petrosal nerve and together forms the vidian nerve. The GSPN is embedded in a meningeal dural sheath while passing on the MCF floor. This meningeal sheath is separable from the endosteal dura of the temporal lobe and this microanatomical nuance is the basis of safely exposing the GSPN while performing a middle fossa approach. The GSPN may be (at least) partially covered by bone along its passage on the middle fossa floor. The geniculate ganglion may be dehiscent in 15%–25% of cases. Also, the proximal portion of the GSPN may have no bony coverage in up to a third of specimens (4, 30). These anatomical observations have led some to advocate for a front-to-back approach to potentially reduce the risk of a facial palsy during a MCF approach (30).

### Lesser petrosal nerve

Three nerves contribute fibers to the LPN: (1) the tympanic branch of the glossopharyngeal nerve (Jacobson's nerve), (2) the nervus intermedius of the facial nerve, and (3) the auricular branch of the vagus nerve (Arnold's nerve). Of note, it might have a communication with the meningeal branch of V3 (31). Jacobson's nerve contributes most fibers to the LPN and arises from the inferior (aka petrous) ganglion of the glossopharyngeal nerve. Entering the tympanic cavity through the inferior tympanic canaliculus of the temporal bone, it forms the tympanic plexus on the medial wall of the tympanic cavity (mainly on the promontory). More superiorly on the promontory, the tympanic plexus converges to a single trunk exiting through the tegmen tympani to emerge on the MCF floor lateral to the facial hiatus and posterior to the tensor tympani muscle. Here, it is joined by a communicating trunk emerging from the geniculate ganglion to form the LPN. The communicating trunk is formed by the union of fibers from the nervous intermedius and Arnold's nerve. The fibers of Arnold's nerve are formed mainly from the superior (aka jugular) ganglion of the vagus nerve but also contain a small contribution from the inferior ganglion of the glossopharyngeal nerve. It then enters the mastoid canaliculus and enters the temporal bone between the jugular foramen and the facial canal to join the mastoid segment of the facial nerve (32). Its fibers, however, travel in a retrograde fashion to exit the geniculate ganglion while being joined by fibers from the nervus intermedius. Fibers from Arnold's nerve convey sensory information from the back of the pinna and the external acoustic meatus.

Once formed, the LPN courses anteriorly on the tensor tympani muscle while being anterior and lateral to the GSPN (see Part 1, Figure 5A). Usually, the nerve is partially uncovered along its course on the MCF floor. The LPN courses an average length of 15 mm on the floor of the middle fossa before piercing the floor and exiting the fossa to join the otic ganglion. As stated in Part 1, the LPN most commonly (70%) exits the MCF through CI. Less frequently, it may exit through the FS or sphenopetrosal suture (32).

## Anatomical–surgical triangles of the MCF

The MCF is a complex anatomic region and understanding its complexities could be facilitated through simplification. Translating the 3D relationships between structures using geometric shapes is one way of approaching this complexity. These have been customarily referred to as “triangles” of the skull base though they do not necessarily represent perfect geometrical trigones. The following discussion only focuses on the MCF triangles and does not include cavernous sinus and posterior fossa triangles.

### Lateral triangle

This triangle is the lateral-most triangle of the MCF. Its arms include the edge of the MCF along the temporal squama laterally, the sphenosquamosal suture anteriorly, and a line between the posterior root of the zygoma and FS posteriorly (see Part 1, Figure 4A). Drilling this bony area, which is technically the roof of the infratemporal fossa, exposes the lateral pterygoid muscle, the pterygoid venous plexus, and the pterygoid segment of IMA, which can be used as a donor for intracranial bypass procedures (33).

### Anteromedial triangle

Named after Sean Mullan who described it first in 1979 and used by him to surgically access the carotid-cavernous fistulas (34), this triangle is simply formed between the V1 and V2. If the greater sphenoid wing is drilled between V1 and V2, the sphenoid sinus will be exposed (see Part 1, Figure 6C).

### Anterolateral triangle

First described as “lateral triangle” by Dolenc, this triangle is formed between V2 and V3 with its anterolateral border being the line connecting FO and FR (35). Other names used to refer to this triangle include “far lateral” and “lateral-most.” The vidian canal passes through the pterygoid process of the sphenoid bone beneath the anterolateral triangle. Drilling the triangle posteriorly exposes the Eustachian tube and drilling it inferomedially exposes the lateral recess of the sphenoid sinus. The infratemporal fossa lies inferior to the anterolateral triangle (see Part 1, Figure 6C).

### The middle fossa rhomboid

Kawase et al. described for the first time an area of the petrous bone not harboring any critical structures that could be removed to access the proximal basilar artery aneurysms (36). This area is essentially the petrous apex and resembles a rhomboid when viewed from an MCF perspective. Its boundaries include the superior semicircular canal (i.e., arcuate eminence) posteriorly, the GSPN laterally, the petrous ridge medially and the Gasserian ganglion anteriorly (see Part 1, Figure 6E). The middle fossa rhomboid could further be divided into premeatal and postmeatal triangles divided by the IAC (Figure 1). The premeatal triangle is essentially the Kawase triangle that could be completely drilled to expose the region between the SPS and inferior petrosal sinus (IPS). Such bony removal would provide better access to the ventrolateral pontomesencephalic and retroclival regions.

### Posterolateral triangle

First described by Glasscock in 1979, this triangle permits access to the petrous ICA through drilling of the MCF just lateral to the GSPN (see Part 1, Figure 5A) (37). Boundaries of this triangle include the lateral border of the V3 distal to the point where GSPN crosses under V3 anteriorly, GSPN medially, and a line from FS to AE laterally (Figure 1). Drilling the triangle just lateral to the GSPN will expose the petrous ICA and the Eustachian tube just lateral to it.

## Surgical approaches^1^

The multiple critical structures present in the vicinity of the MCF make for a suitable corridor and/or target for various pathologies within its anatomic region. Surgical approaches to the MCF date back to the late 19th century with the extradural subtemporal approach first described by Frank Hartley (1856–1913) and Fedor Krause (1857–1937)—later eponymously known as the Hartley–Krause procedure. Their described approach was used to treat trigeminal neuralgia *via* a Gasserian ganglionectomy—the nerves were divided at FR and FO and excised to a point back beyond the Gasserian ganglion. In 1900, Harvey Cushing (1869–1939) modified the procedure by using a more basal trajectory to minimize brain retraction—his contribution decreased the mortality rate to 5% (38). Later, in 1904, Parry described the middle fossa approach for sectioning the vestibular nerve (39).

### Middle fossa and extended middle fossa approaches

In 1975, Bochenek and Kukwa modified the subtemporal approach to improve the exposure of ventrolateral pons and cerebellopontine angle down to the region of the jugular foramen. They called this approach the extended middle fossa approach (40). Their modification included the drilling of the petrous bone. By doing this, 10 mm of extra space could be gained below the level of the SPS, allowing better access to the basilar artery. The major disadvantage of this modified approach is damage to hearing function due to the damage to the bony labyrinth (i.e., drilling the semicircular canals).

**Figure 8:**
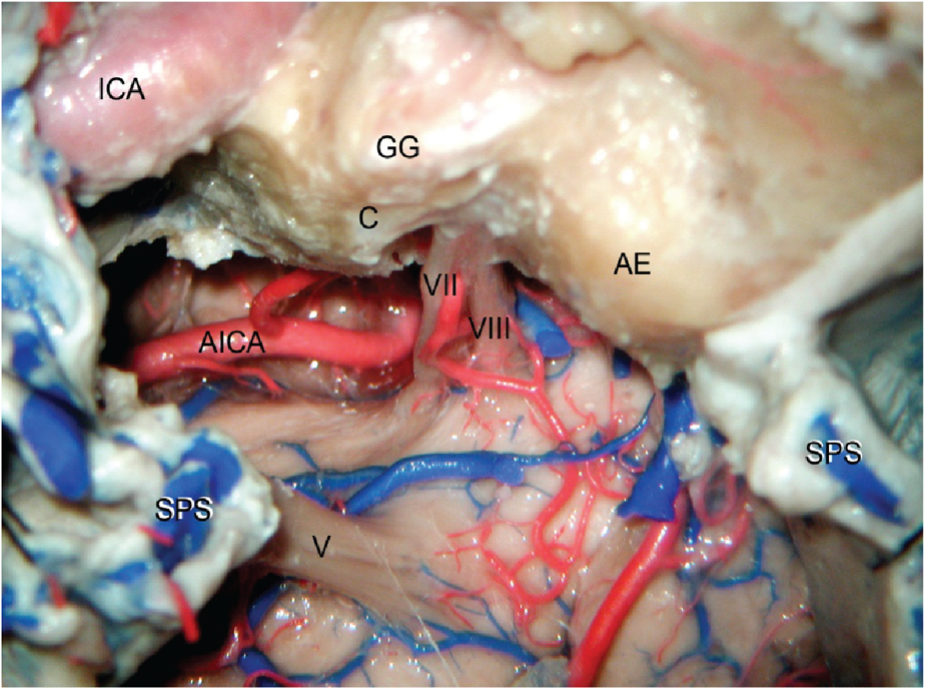
Exposure of the ventrolateral aspect of the pons using right anterior petrosectomy (also known as Kawase's approach). A complete resection of the petrous apex is undertaken including the premeatal and postmeatal triangles. The cochlea (C) and arcuate eminence (AE) are preserved and the carotid canal is unroofed to expose the petrous ICA. Note how sectioning the SPS allows exposure of the entire ventrolateral aspect of the pons between the cranial nerves V and VII–VIII. ICA, internal carotid artery; SPS, superior petrosal sinus; AICA, anterior inferior cerebellar artery; GG, geniculate ganglion. (From Zhao and Liu. *Neurosurg Focus*. 2008 **25**(6): E5. Used with permission from JNS Publishing Group.)

Later, in 1985, the “extended middle fossa approach” was further modified by Takeshi Kawase—now eponymously called the Kawase approach. Specifically, Kawase designed this approach to treat petroclival meningiomas extending into the parasellar region (i.e., sphenopetroclival meningiomas) (41). Here, in comparison to the modification of Bochenek and Kukwa, only the petrous apex is drilled. Selective drilling of the apex is advantageous because it contains no neurovascular structures while providing excellent exposure of the ventrolateral brainstem (Figure 8). In addition, the approach has an extra step: transverse division of the tentorium to the incisura. This extra maneuver allows communication between the intradural middle and posterior cranial fossae.

**Figure 9:**
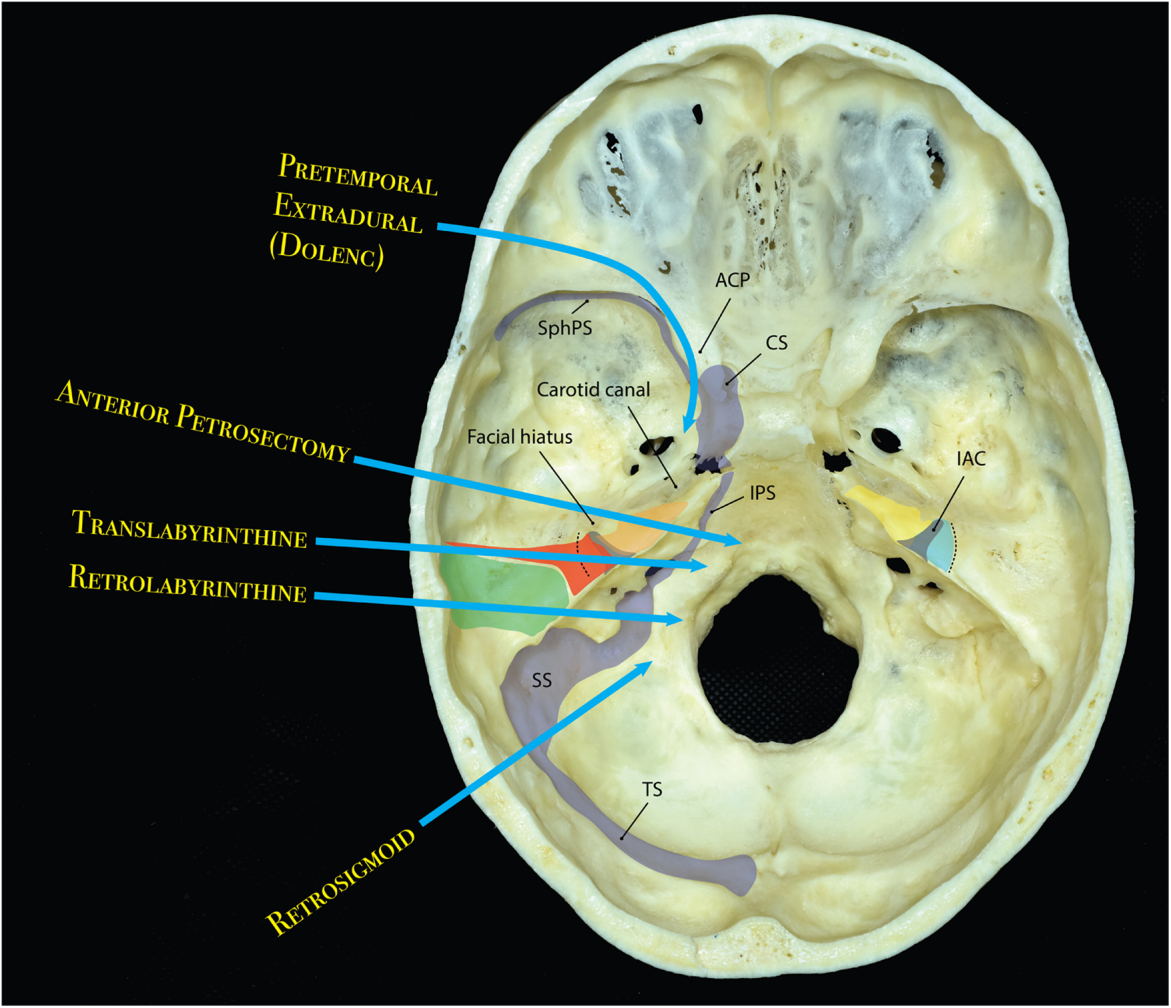
Diagram showing different approaches and their approximate areas of bony resection and trajectory to the central skull base and posterior fossa through the middle cranial fossa. Left: anterior petrosectomy (orange) can be combined with graded increments of posterior petrosectomy; i.e., retrolabyrinthine (green) and translabyrinthine (red). Right: anterior petrosectomy (yellow), may also by extended posteriorly by drilling the roof of the IAC hence adding a postmeatal extension (blue). IAC is shown as a gray shaded funnel bilaterally. Dotted line shows the approximate area of arcuate eminence. ACP, anterior clinoid process; CS, cavernous sinus; IAC, internal auditory canal; IPS, inferior petrosal sinus; SphPS, sphenoparietal sinus; SS, sigmoid sinus; TS, transverse sinus. (Copyright Ali Tayebi Meybodi. Used with permission)

**Figure 10:**
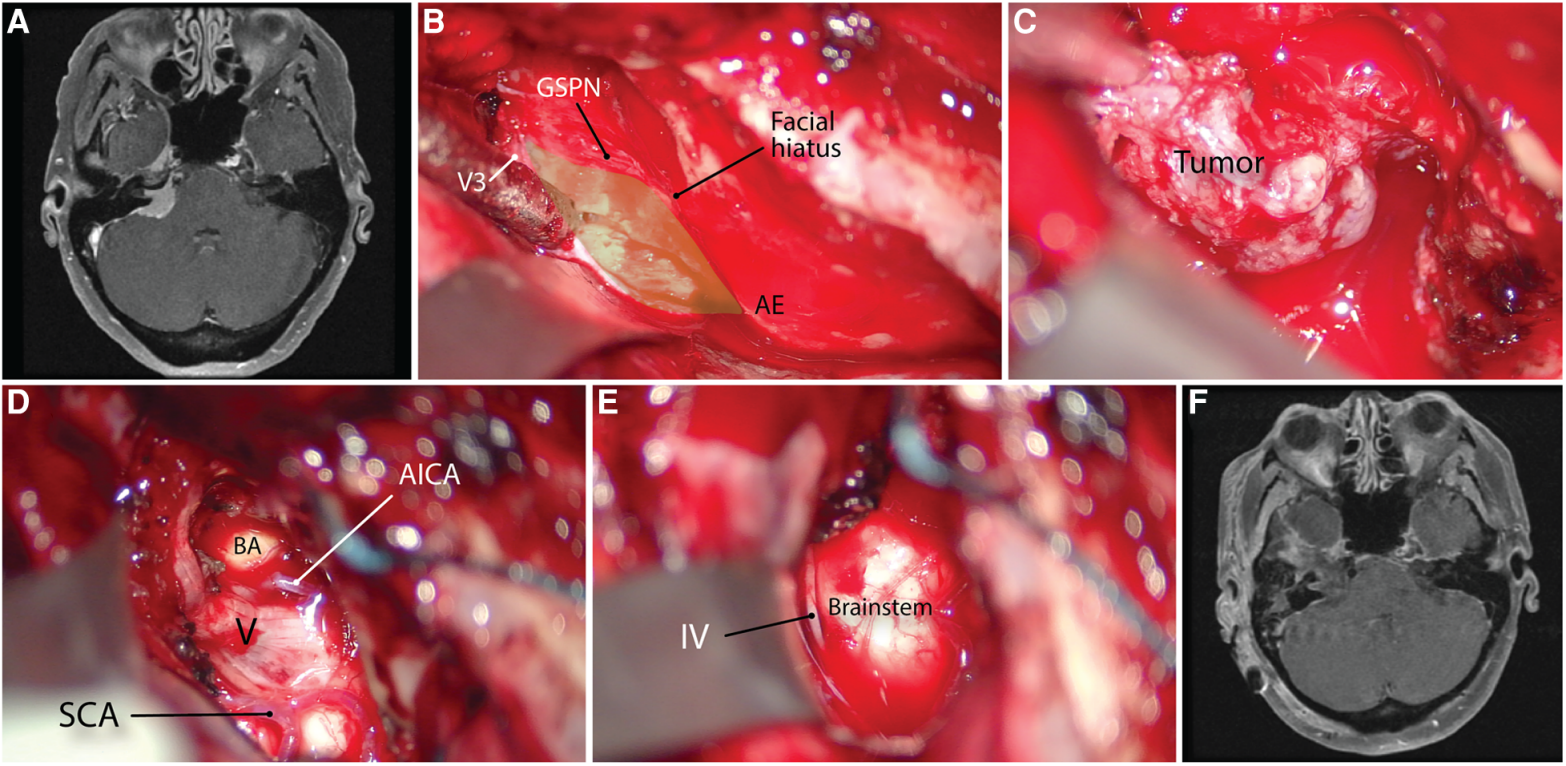
Using the anterior petrosectomy approach for petrous apex meningioma resection. (**A**) Preoperative axial contrasted MRI scan. (**B**) Exposure of the right middle fossa floor and the middle fossa rhomboid (green area) using the anatomic landmarks. (**C**) exposure of the tumor. (**D,E**) Post-resection cavity showing the exposure of the brainstem and trigeminal nerve. (**F**) Postoperative contrasted MRI scan showing gross total resection of tumor. AICA, anterior inferior cerebellar artery; AE, arcuate eminence; BA, basilar artery; GSPN, greater superficial petrosal nerve; IV, trochlear nerve, SCA, superior cerebellar artery. V, trigeminal nerve. (Copyright James K. Liu. Used with permission.)

**Figure 11:**
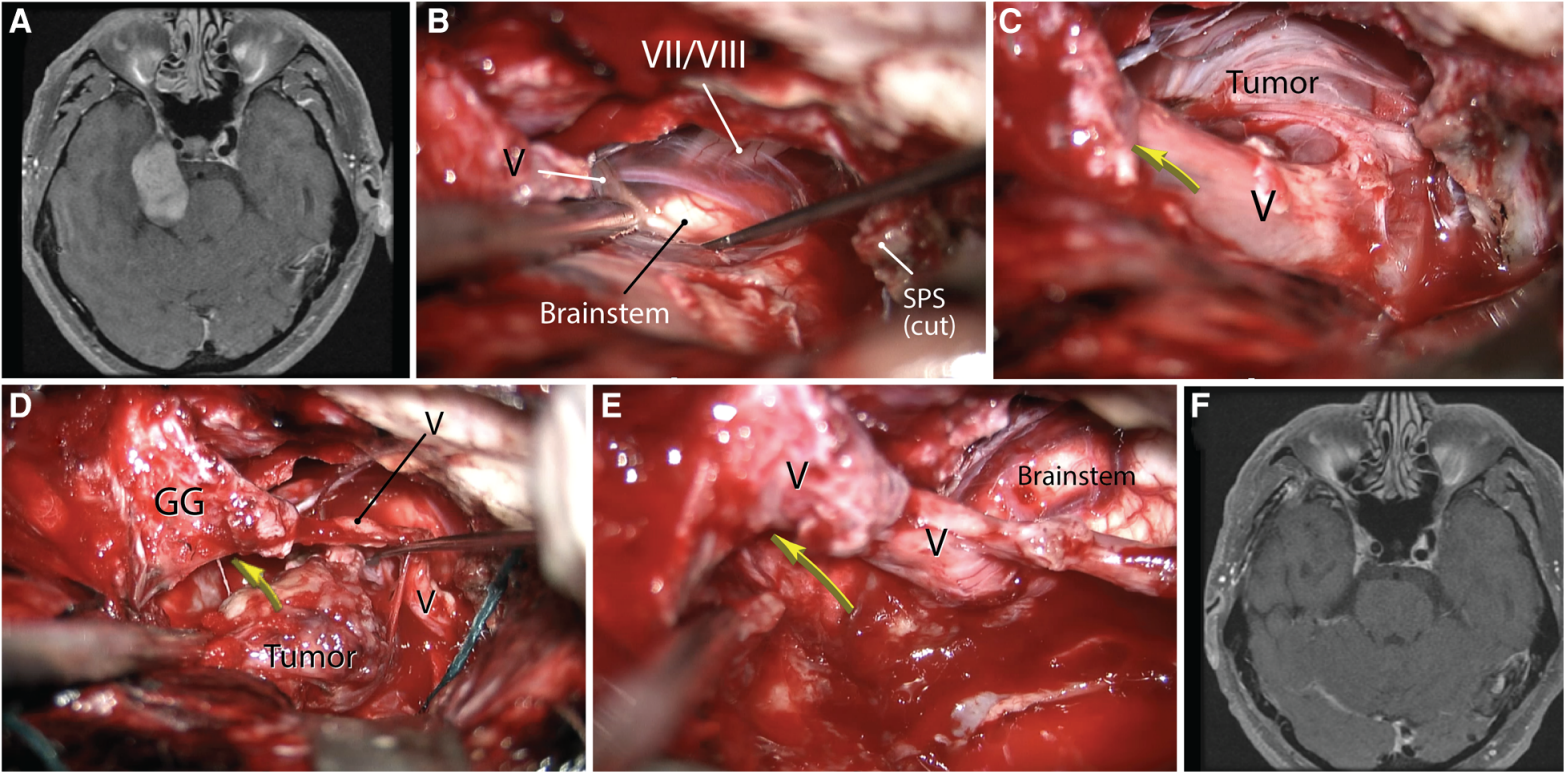
Using the anterior petrosectomy approach for a right-sided dumbbell-shaped trigeminal schwannoma (**A**). (**B–E**) This approach allows exposure of cranial nerves V and VII/VIII as well as Meckel's cave (yellow arrow) and complete tumor resection. (**F**) Postoperative contrasted MRI. GG, Gasserian ganglion, SPS, superior petrosal sinus, V, trigeminal nerve, VII/VIII, facial-acoustic bundle. (Copyright James K. Liu. Used with permission.)

**Figure 12:**
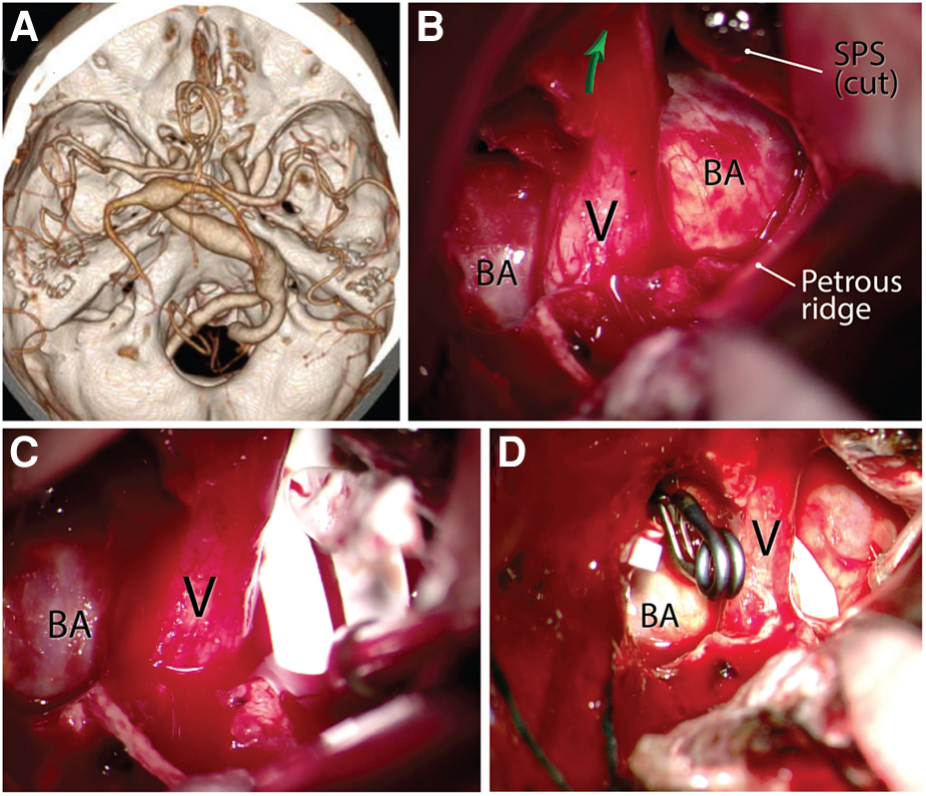
Using the anterior petrosectomy approach for macrovascular decompression of the left trigeminal nerve for intractable trigeminal neuralgia caused by a dolichoectatic basilar artery (**A**). (**B**) Left anterior petrosectomy showing the exposure of the pathology. Green arrow shows the orifice of the Meckel's cave. (**C**) A sling is placed around the offending basilar artery in the shape of a lasso. (**D**) Clip transposition and arteriopexy of the dolichoectatic basilar artery to the adjacent skull base dura away from the trigeminal nerve after application of the sling. BA, basilar artery; SPS, superior petrosal sinus; V, trigeminal nerve. (Courtesy of Barrow Neurological Institute, Phoenix, AZ, United States. Used with permission.)

The extradural middle fossa approach is also used to treat a variety of other pathologies such as petrous apex lesions, intracanalicular vestibular schwannomas, exposure of the petrous ICA as a donor for cerebral bypass procedures, and access to the infratemporal fossa to treat pathologies or to expose the IMA as a bypass donor. More recently, the term “extended middle fossa approach” is used when drilling of the MCF floor is not restricted to the petrous apex and unroofing the IAC. Basically, the surgeon could extend the drilling to the postmeatal triangle between the arcuate eminence and IAC to improve exposure of the contents of the IAC. Additionally, a limited mastoidectomy could be added if necessary (42). A full mastoidectomy with graded increase in labyrinthectomy (i.e., posterior petrosectomy) can be combined with anterior petrosectomy to provide an excellent exposure of large petroclival lesions (Figure 9) (43, 44). Further removal of the cochlea and mobilization of the zygoma will increase the surgical exposure of and maneuverability on the ventrolateral pontomesencephalic area (45). The details of these surgical maneuvers are beyond the scope of this discussion. Figures 10–12 provide case illustrations.

### Subtemporal approach

In 1961, the Canadian neurosurgeon, Charles Drake (1920–1998), introduced a new surgical approach to the medial portion of the MCF called the subtemporal approach—designed for posterior circulation aneurysms (46). It is classically described as a 5 cm × 5 cm craniotomy located below the superior temporal line and centered in the coronal plane above the root of the zygoma. It requires drilling of the temporal bone to the floor of the middle fossa. The subtemporal approach is an intradural approach and the surgeon should be aware of the temporobasal veins draining the lateral parts of the basal surface of the temporal lobe into the lateral tentorial sinuses. Also, the vein(s) of Labbé draining into the transverse-sigmoid junction should be preserved.

### Endonasal approaches to the middle fossa

Influential advances in endoscopic skull base surgery have validated these approaches as reasonably less invasive alternatives to the “traditional” open lateral approaches. Using the expanded corridors of the nasal and orbital cavities, the surgeon could practically access the anteromedial regions of the MCF without the need for retraction of the temporal lobe (47). Such approaches may be used for a variety of pathologies including but not limited to trigeminal neuromas, Meckel's cave meningiomas, chordomas and chondrosarcomas, juvenile angiofibromas, and other petrous apex lesions such as cholesterol granulomas. The final exposure arena will include the anteromedial temporal lobe dura posterior to the pterygopalatine fossa and the greater wing of the sphenoid, Gasserian ganglion, and the maxillary and mandibular nerves. Essentially, the endonasal approach involves a transpterygoid approach and the lateral aspect of the sphenoid sinus to expose the so-called “quadrangular space” lateral to the paraclival ICA and inferior to the abducens nerve (48, 49).

Transorbital endoscopic skull base approaches are also gaining popularity because of the direct access to the MCF. Through exposure of the frontosphenoid suture and the greater sphenoid wing, dura of the temporal pole, and anterior MCF could be exposed (50). Detailed discussion on the surgical anatomy of the endoscopic approaches is beyond the scope of this piece, and the reader is referred to the many available excellent studies (47, 51, 52).

## Conclusion

A detailed understanding of the MCF microanatomy is required to successfully navigate it without complications. The present review tries to encompass most of the relevant neurovascular anatomic nuances that are of practical significance when approaching the MCF. It should be noted, however, that the surgical approaches need to be studied and practiced in a cadaver lab to maximize the application of the anatomic facts during surgery. Also, many pathologic states alter the normal anatomical relationships and, therefore, each surgical approach has to be specifically tailored to the individual patient pathoanatomy.

## Footnotes

^1^Although infratemporal and pterygopalatine fossa are located on the exocranial aspect of the middle cranial fossa, they are not discussed here as their anatomy and distinct surgical importance require dedicated detailed discussions.

## References

[1] Osawa S Rhoton AL Jr. Tanriover N Shimizu S Fujii K. Microsurgical anatomy and surgical exposure of the petrous segment of the internal carotid artery. Neurosurgery. (2008) 63(4 Suppl 2):210–38, discussion 239. 10.1227/01.NEU.0000327037.75571.10 18981828

[2] Morris H Lond MB Playfair McMurrich J, editors.Morris’s human anatomy. A complete systematic treatise by English and American authors. 5th ed. Philadelphia, PA: P. Blakiston’s Son & Co (1907).

[3] Bradač GB. Applied cerebral angiography: Normal anatomy and vascular pathology. 3rd ed. Berlin: Springer (2017).

[4] Paullus WS Pait TG Rhoton AI Jr. Microsurgical exposure of the petrous portion of the carotid artery. J Neurosurg. (1977) 47(5):713–26. 10.3171/jns.1977.47.5.0713 908935

[5] Steffen TN. Vascular anomalites of the middle ear. Laryngoscope. (1968) 78(2):171–97. 10.1288/00005537-196802000-00001 5644220

[6] Silbergleit R Quint DJ Mehta BA Patel SC Metes JJ Noujaim SE. The persistent stapedial artery. AJNR Am J Neuroradiol. (2000) 21(3):572–7. PMID: ; PMCID: 10730654 PMC8174972

[7] Altmann F. Anomalies of the internal carotid artery and its branches; their embryological and comparative anatomical significance; report of a new case of persistent stapedial artery in man. Laryngoscope. (1947) 57(5):313–39. 10.1288/00005537-194705000-00002 20241850

[8] Yoshida K Akiyama T Raz E Kamamoto D Ozawa H Toda M. Angio-anatomical study of the pterygovaginal artery based on cone-beam computed tomography. Neuroradiology. (2021) 63(8):1325–33. 10.1007/s00234-021-02657-3 33555352

[9] El-Khouly H Fernandez-Miranda J Rhoton AL Jr. Blood supply of the facial nerve in the middle fossa: the petrosal artery. Neurosurgery. (2008) 62(5 Suppl 2):ONS297–303, discussion ONS303–294. 10.1227/01.neu.0000326010.53821.a3 18596507

[10] Martins C Yasuda A Campero A Ulm AJ Tanriover N Rhoton A. Microsurgical anatomy of the dural arteries. Neurosurgery. (2005) 56(2 Suppl):211–51, discussion 211–51. 10.1227/01.neu.0000144823.94402.3d 15794820

[11] Breschet G. Recherches anatomiques, physiologiques et pathologiques sur le systéme veineux et spécialement sur les canaux veineux des Os. Paris: Villeret et Rouen (1829). p. 1–42.

[12] San Millan Ruiz D Fasel JH Rufenacht DA Gailloud P. The sphenoparietal sinus of breschet: does it exist? An anatomic study. AJNR Am J Neuroradiol. (2004) 25(1):112–20. PMID: ; PMCID: 14729539 PMC7974157

[13] Shibao S Toda M Orii M Fujiwara H Yoshida K. Various patterns of the middle cerebral vein and preservation of venous drainage during the anterior transpetrosal approach. J Neurosurg. (2016) 124(2):432–9. 10.3171/2015.1.JNS141854 26314997

[14] Rhoton AL Jr. The cerebral veins. Neurosurgery. (2002) 51(4 Suppl):S159–205. 10.1097/00006123-200210001-00005 12234449

[15] Simoes S. An anatomical study of the laterotrigeminal venous system. Ann Anat. (1993) 175(2):115–8. 10.1016/S0940-9602(11)80163-9 8489031

[16] Matsushima K Matsushima T Kuga Y Kodama Y Inoue K Ohnishi H Classification of the superior petrosal veins and sinus based on drainage pattern. Neurosurgery. (2014) 10(Suppl 2):357–67, discussion 367. 10.1227/NEU.0000000000000323 24561869

[17] Tubbs RS Mortazavi MM Krishnamurthy S Verma K Griessenauer CJ Cohen-Gadol AA. The relationship between the superior petrosal sinus and the porus trigeminus: an anatomical study. J Neurosurg. (2013) 119(5):1221–5. 10.3171/2013.4.JNS122062 23706047

[18] Coates AE. A note on the superior petrosal sinus and its relation to the sensory root of the trigeminal nerve. J Anat. (1934) 68(Pt 3):428. PMID: ; PMCID: 17104494 PMC1249045

[19] Tanriover N Abe H Rhoton AL Jr. Kawashima M Sanus GZ Akar Z. Microsurgical anatomy of the superior petrosal venous complex: new classifications and implications for subtemporal transtentorial and retrosigmoid suprameatal approaches. J Neurosurg. (2007) 106(6):1041–50. 10.3171/jns.2007.106.6.1041 17564177

[20] Rhoton AL. The cavernous sinus, the cavernous venous plexus, and the carotid collar. Neurosurgery. (2002) 51(4 Suppl):S375–410. 10.1097/00006123-200210001-00010 12234454

[21] Joo W Yoshioka F Funaki T Mizokami K Rhoton AL. Microsurgical anatomy of the trigeminal nerve. Clin Anat. (2014) 27(1):61–88. 10.1002/ca.22330 24323792

[22] Natori Y Rhoton AL. Microsurgical anatomy of the superior orbital fissure. Neurosurgery. (1995) 36(4):762–75. 10.1227/00006123-199504000-00018 7596508

[23] Shi X Han H Zhao J Zhou C. Microsurgical anatomy of the superior orbital fissure. Clin Anat. (2007) 20(4):362–6. 10.1002/ca.20391 17080461

[24] Shankland WE 2nd. The trigeminal nerve. Part IV: the mandibular division. Cranio. (2001) 19(3):153–61. 10.1080/08869634.2001.11746164 11482826

[25] Shankland WE 2nd. The trigeminal nerve. Part III: the maxillary division. Cranio. (2001) 19(2):78–83. 10.1080/08869634.2001.11746155 11842868

[26] Martinez Pascual P Maranillo E Vazquez T Simon de Blas C Lasso JM Sanudo JR. Extracranial course of the facial nerve revisited. Anat Rec. (2019) 302(4):599–608. 10.1002/ar.23825 29659175

[27] Gardner WJ Stowell A Dutlinger R. Resection of the greater superficial petrosal nerve in the treatment of unilateral headache. J Neurosurg. (1947) 4(2):105–14. 10.3171/jns.1947.4.2.0105 20293608

[28] Ginsberg LE De Monte F Gillenwater AM. Greater superficial petrosal nerve: anatomy and MR findings in perineural tumor spread. AJNR Am J Neuroradiol. (1996) 17(2):389–93. PMID: ; PMCID: 8938317 PMC8338384

[29] Tubbs RS Menendez J Loukas M Shoja MM Shokouhi G Salter EG The petrosal nerves: anatomy, pathology, and surgical considerations. Clin Anat. (2009) 22(5):537–44. 10.1002/ca.20814 19544297

[30] Jittapiromsak P Sabuncuoglu H Deshmukh P Nakaji P Spetzler RF Preul MC. Greater superficial petrosal nerve dissection: back to front or front to back? Neurosurgery. (2009) 64(5 Suppl 2):253–8, discussion 258–259. 10.1227/01.NEU.0000343522.79764.15 19404106

[31] Kakizawa Y Abe H Fukushima Y Hongo K El-Khouly H Rhoton AL Jr. The course of the lesser petrosal nerve on the middle cranial fossa. Neurosurgery. (2007) 61(3 Suppl):15–23, discussion 23. 10.1227/01.neu.0000289707.49684.a3 17876229

[32] Tekdemir I Aslan A Elhan A. A clinico-anatomic study of the auricular branch of the vagus nerve and Arnold’s ear-cough reflex. Surg Radiol Anat. (1998) 20(4):253–7. 10.1007/s00276-998-0253-5 9787391

[33] Feng X Lawton MT Rincon-Torroella J El-Sayed IH Meybodi AT Benet A. The lateral triangle of the middle fossa: surgical anatomy and a novel technique for transcranial exposure of the internal maxillary artery. Oper Neurosurg. (2016) 12(2):106–11. 10.1227/NEU.0000000000001099 29506088

[34] Mullan S. Treatment of carotid-cavernous fistulas by cavernous sinus occlusion. J Neurosurg. (1979) 50(2):131–44. 10.3171/jns.1979.50.2.0131 430123

[35] Dolenc VV. General approach to the cavernous sinus. In: Anatomy and surgery of the cavernous sinus. Vienna: Springer Vienna (1989). p. 139–69.

[36] Kawase T Toya S Shiobara R Mine T. Transpetrosal approach for aneurysms of the lower basilar artery. J Neurosurg. (1985) 63(6):857–61. 10.3171/jns.1985.63.6.0857 4056899

[37] Glassock ME 3rd Jackson CG Dickins JR Wiet RJ. Panel discussion: glomus jugulare tumors of the temporal bone. The surgical management of glomus tumors. Laryngoscope. (1979) 89(10 Pt 1):1640–54. 10.1002/lary.5540891015 228135

[38] Cole CD Liu JK Apfelbaum RI. Historical perspectives on the diagnosis and treatment of trigeminal neuralgia. Neurosurg Focus. (2005) 18(5):E4. 10.3171/foc.2005.18.5.5 15913280

[39] Parry RH. A case of tinnitus and vertigo treated by division of the auditory nerve. J Laryngol Otol. (1991) 105(12):1099–100. 10.1017/S0022215100118328 1787370

[40] Bochenek Z Kukwa A. An extended approach through the middle cranial fossa to the internal auditory meatus and the cerebello-pontine angle. Acta Otolaryngol. (1975) 80(5–6):410–4. 10.3109/00016487509121344 1081810

[41] Kawase T Shiobara R Toya S. Anterior transpetrosal-transtentorial approach for sphenopetroclival meningiomas: surgical method and results in 10 patients. Neurosurgery. (1991) 28(6):869–75, discussion 875–76. 10.1227/00006123-199106000-00014 2067611

[42] Liu JK. Extended middle fossa approach with anterior petrosectomy for resection of upper petroclival meningioma involving Meckel’s cave: operative video and technical nuances. Neurosurg Focus. (2017) 43(Videosuppl 2):V8. 10.3171/2017.10.FocusVid.17345 28967313

[43] Meybodi AT Liu JK. Endoscopic-assisted combined transcrusal anterior petrosal approach for resection of large petroclival meningioma: operative video and nuances of technique. Neurosurg Focus Video. (2022) 6(2):V10. 10.3171/2022.1.FOCVID21257 PMC9559220 36285004

[44] Polster SP Horowitz PM Awad IA Gluth MB. Combined petrosal approach. Curr Opin Otolaryngol Head Neck Surg. (2018) 26(5):293–301. 10.1097/MOO.0000000000000480 30045103

[45] Hsu FP Anderson GJ Dogan A Finizio J Noguchi A Liu KC McMenomey SO Extended middle fossa approach: quantitative analysis of petroclival exposure and surgical freedom as a function of successive temporal bone removal by using frameless stereotaxy. J Neurosurg. (2004) 100(4):695–9. 10.3171/jns.2004.100.4.0695 15070125

[46] Drake CG. Bleeding aneurysms of the basilar artery. Direct surgical management in four cases. J Neurosurg. (1961) 18:230–8. 10.3171/jns.1961.18.2.0230 13724254

[47] Prevedello DM Ditzel Filho LF Solari D Carrau RL Kassam AB. Expanded endonasal approaches to middle cranial fossa and posterior fossa tumors. Neurosurg Clin N Am. (2010) 21(4):621–35, vi. 10.1016/j.nec.2010.07.003 20947031

[48] Kassam AB Prevedello DM Carrau RL Snyderman CH Gardner P Osawa S The front door to Meckel’s cave: an anteromedial corridor via expanded endoscopic endonasal approach—technical considerations and clinical series. Neurosurgery. (2009) 64(3 Suppl):ons71–82, discussion ons82–73. 10.1227/01.NEU.0000335162.36862.54 19240575

[49] Saraceno G Agosti E Qiu J Buffoli B Ferrari M Raffetti E Quantitative anatomical comparison of anterior, anterolateral and lateral, microsurgical and endoscopic approaches to the middle cranial Fossa. World Neurosurg. (2020) 134:e682–730. 10.1016/j.wneu.2019.10.178 31731015

[50] Chibbaro S Ganau M Scibilia A Todeschi J Zaed I Bozzi MT Endoscopic transorbital approaches to anterior and middle cranial fossa: exploring the potentialities of a modified lateral retrocanthal approach. World Neurosurg. (2021) 150:e74–80. 10.1016/j.wneu.2021.02.095 33647487

[51] Guizzardi G Mosteiro A Hoyos J Ferres A Topczewski T Reyes L Endoscopic transorbital approach to the middle fossa: qualitative and quantitative anatomic study. Oper Neurosurg. (2022) 23(4):e267–75. 10.1227/ons.0000000000000308 36106937

[52] de Lara D Ditzel Filho LF Prevedello DM Carrau RL Kasemsiri P Otto BA Endonasal endoscopic approaches to the paramedian skull base. World Neurosurg. (2014) 82(6 Suppl):S121–9. 10.1016/j.wneu.2014.07.036 25496623

